# Editorial for the Beyond Moore’s Law: Hardware Specialization and Advanced System on Chip

**DOI:** 10.3390/mi14081583

**Published:** 2023-08-11

**Authors:** Xiaokun Yang

**Affiliations:** College of Science and Engineering, University of Houston Clear Lake, Houston, TX 77058, USA; yangxia@uhcl.edu

In the absence of a new transistor technology to replace CMOS, design specialization has emerged as one of the most immediate options for achieving high-performance computing. One notable example of purpose-built architecture for inference workloads is the Google tensor processing unit (TPU). The TPU has demonstrated significantly higher efficiency compared to using a general-purpose chip. In the field of artificial intelligence, there have been numerous successful implementations of application-specific designs and accelerators in industry. For instance, Nervana’s AI architecture, Facebook’s “Big Sur”, and various forms of computer-in-network acceleration for large data centers, such as Microsoft’s FPGA Configurable Cloud and Project Catapult for FPGA-accelerated search. The objective of this Special Issue is to explore a wide range of research and demonstrations on computation-intensive applications for high-performance computing, focusing on the various specialized designs that have been developed.

Specifically, Sha et al. (reference [1]) present a design structure that integrates a CPU-based control plane and an FPGA-based data plane. Their aim is to support multiple network functions while achieving a high performance at 100 Gbps. In the deep learning field, Xu et al. (reference [2]) propose a low-power design for the YOLOv4-tiny model using an FPGA. Their design utilizes 16-bit fixed-point operators, which trade precision for the achievement of over 10 times and 3 times the power dissipation compared to CPU and GPU, respectively. Another paper in the neural network design field is from Xie et al. (reference [3]), who demonstrate an efficient accelerator for N:M sparse convolutional neural networks (CNNs) with layer-wise sparse patterns. Their implementation of FPGA validates the acceleration of classical CNNs such as Alexnet, VGG-16, and ResNet-50. Madineni et al. (reference [4]) present a parameterized design of a CNN network using Chisel, an open-source hardware construction language developed at UC Berkeley. This design allows for flexible implementation options, supporting 16-bit, 32-bit, 64-bit, and 128-bit configurations on FPGA. Popovici et al. (reference [5]) introduce a real-time RISC-V-based CAN-FD bus diagnosis tool and make the design publicly available. Wang et al. (reference [6]) propose a TCP offload engine (TOE) prototype system on an FPGA to support a 100 Gbps high-performance throughput. Their design enables the concurrent processing of from hundreds to 250,000 TCP connection state hardware maintenance on a single network node, thereby improving the overall performance of the network system.
